# Calculating unreported confidence intervals for paired data

**DOI:** 10.1186/1471-2288-11-66

**Published:** 2011-05-12

**Authors:** Karim F Hirji, Morten W Fagerland

**Affiliations:** 1 Department of Epidemiology and Biostatistics, Muhimbili University of Health and Allied Sciences, Dar es Salaam, Tanzania; 2 Unit of Biostatistics and Epidemiology, Oslo University Hospital, N-0407 Oslo, Norway

## Abstract

**Background:**

Confidence intervals (or associated standard errors) facilitate assessment of the practical importance of the findings of a health study, and their incorporation into a meta-analysis. For paired design studies, these items are often not reported. Since the descriptive statistics for such studies are usually presented in the same way as for unpaired designs, direct computation of the standard error is not possible without additional information.

**Methods:**

Elementary, well-known relationships between standard errors and *p*-values were used to develop computation schemes for paired mean difference, risk difference, risk ratio and odds ratio.

**Results:**

Unreported confidence intervals for large sample paired binary and numeric data can be computed fairly accurately using simple methods provided the *p*-value is given. In the case of paired binary data, the design based 2 × 2 table can be reconstructed as well.

**Conclusions:**

Our results will facilitate appropriate interpretation of paired design studies, and their incorporation into meta-analyses.

## Background

Not too long ago, analyses of research data comprised performing a large number of unplanned hypothesis tests, and reporting the results simply as *p *< 0.05 or not, often in a selective way. Described as the cult of statistical significance, this practice frequently produced flawed interpretations, and made judging their practical importance difficult. Extensive criticism has now put it out of favor [1]. Reporting guidelines and many journals currently require joint presentation of the three key statistical indices, namely, point estimate, actual *p*-value, and confidence interval (CI), especially for the principal analyses of the study [2,3]. The CI (or associated standard error) enables interpretation of the main effect measure in a more complete manner, and is needed for incorporating the study results into a meta-analysis.

Various remedies for different instances of incomplete or varied reporting of essential findings (like confidence intervals) and key data have been proposed. For independent-groups 2 × 2 tables, Pietrantonj [4] detailed and evaluated a series of methods for reconstructing the unreported data table in risk difference-, risk ratio-, and odds ratio-based analyses. For a factor variable presented in quantile groups, Chêne and Thompson [5] described how findings from diverse forms of analyses can be re-expressed in terms of the mean difference. Abrams et al. [6] looked at clinical trials reporting quantitative change from baseline and gave Bayesian and sensitivity analyses methods to address partial reporting. Focusing on two-period cross-over trials, Elbourne et al. [7] presented simple formulas linking the relevant standard deviation and correlation, for both continuous and binary data. These methods can be used to estimate one when the other is given. Tang et al. [8] described and evaluated methods to find the variance of the difference between paired proportions using a specialized variance recovery method that utilizes the variances of the individual proportions.

Our paper is located within this broad field dealing with incomplete reporting of data or analysis results. We focus on pair matched designs, specifically matched and cross-over clinical trials, paired cohort studies, and 1:1 matched case-control studies. We first note that for such studies, confidence interval-related reporting shortfalls persist. Mills et al. [9] scrutinized a sample of 526 randomised controlled trials. Among these, 116 or 22% were crossover trials. Of the latter, only 30% presented a CI or the standard error, 62% did not present a CI but gave enough information to enable its computation, and the remaining 8% did not report it and did not give data enabling its computation. Their paper, however, does not specify how the computability of the CI was judged. Poor reporting in cross-over trials was also underscored by Elbourne et al. [7].

We assessed the current state of CI reporting in paired epidemiologic studies, albeit in a preliminary way, with a systematic survey of the PubMed database. It was searched on April 16, 2010 using the term "matched case-control study", then sorted by "Recently Added". Studies that classified themselves in the abstract as 1:1 matched case-control studies were potentially eligible. If further scrutiny revealed the actual matching not to be 1:1, the study was excluded. The first 20 studies satisfying our criteria were selected. The median number of pairs in these studies was 196 (range 7 to 42 542 pairs), and one fifth had fewer than 50 pairs. The full list of these studies is available from the authors.

Twelve studies were true case-control studies and eight had a pair matched design but it was not a case-control type. All of the 12 true case-control studies reported CIs, but three used inappropriate unpaired data methods. Only two of the other eight studies used paired data methods. None of them reported a CI, but seven gave sufficient data to compute it. Overall, in the twenty self-labeled pair matched case-control studies, correct calculation and reporting of CI was done in 9/20 (45%), and a correct CI was computed or was directly computable in 16/20 (80%).

When a paper lacks a CI or the relevant standard error, one option is to contact the authors. If that does not yield the needed data, or if the process is too time-consuming, is there an alternative? Our paper presents simple methods, hitherto underutilized or unpublished, that can be used for this purpose.

## Methods

It is helpful to note that the prevalent style of reporting descriptive statistics makes the problem of non-computability of CIs more acute for paired designs than for independent samples designs. A comparison of two proportions usually reports the sample proportions and sample sizes, and of two means, the sample means, sizes and standard deviations, whether the design is unpaired or paired. For the former design, these quantities respectively suffice to compute the CIs for effect measures like risk difference, risk ratio, odds ratio or the difference of means. In the case of paired data, they do not. For paired binary data, we need the 2 × 2 table with the concordant and discordant pairs, and for paired continuous data, we need the mean difference and standard deviation of the differences. These entities, in part or full, are rarely reported, even when they were used to compute a p-value via appropriate tests like the McNemar's test, paired *t*-test, paired *z*-test, or a paired exact test. If only group level proportions (or means and standard deviations) are given, additional measures, such as between-group correlation, are required to determine the relevant standard error [7,8]. Such entities are hardly reported.

We deal with four common effect measures (mean difference, risk difference, risk ratio and odds ratio) for paired design studies where the CI has not been given, and the design based 2 × 2 table or the standard deviation of the differences (as the case may be) and the relevant correlation are also unknown. For each measure, we show that if the *p*-value is known, the corresponding standard error and CI can be obtained from a simple computation scheme. In the case of binary data, the data table with the concordant and discordant pairs can be reconstructed as well.

## Results

We first introduce the main example we use to illustrate our methods. Xie et al. [10] reported a multi-center study of critically ill surgical patients with severe sepsis (SS). Its main aim was to assess if the presence of invasive fungal infection (IFI) affected the outcome for such cases. The subjects were drawn from the surgical intensive care units of ten teaching hospitals in China. All admissions in the one year study period meeting the set criteria for sepsis were included. The data on patient characteristics, treatments, and outcomes were compiled through daily chart reviews and physician interviews.

The main study had 90 SS patients with IFI and 228 SS patients without IFI. In a sub-study, 60 of the SS patients with IFI were matched, on a one-to-one basis and in terms of center, sex, age and APACHE II score, with 60 SS patients without IFI. We consider the matched portion of this study, and show a part of the results in Table 1.

**Table 1:** Fungal infection and severe sepsis

	Patient group		
**Outcome**	**SS & IFI**	**SS & no IFI**	***p*-value**

Hospital LOS (mean days)	30	20	0.020
Hospital mortality (%)	70.0%	50.0%	0.023
Total subjects	60	60	

Note: LOS = Length of stay. Source: Xie et al. [10], Table five.

### Paired continuous data

One variable (hospital LOS) in Table 1 is continuous; the other (mortality) is binary. For now, consider the former. The paper reports the group interquartile ranges for hospital LOS but not the standard deviations. Also, the CI for the difference in hospital LOS is not given. However, the *p*-value is stated with two significant digits. Using this, we impute the needed CI as follows.

For comparing hospital LOS, the two sided *p *= 0.020. The associated standard normal deviate is *z *= 2.326. In general, let  be the mean of the differences, and *s_z_*, the standard normal paired two-sided *z*-test standard error. The mean of the differences is the difference of the means, so . From the *p*-value, we get *z*, the corresponding two-sided deviate of the standard normal distribution. Then we apply the relation

The 95% CI for the difference of the means thereby is

Suppose the paired two-sided *t*-test was used. From the *p*-value, we get *t*, the corresponding deviate of the *t *distribution with *n *- 1 degrees of freedom. With *s_t _*as the corresponding standard error for the difference of the means, we have

The 95% CI for the difference of the means is then

where *t*_*n *- 1 _is the 97.5th percentile of the *t *distribution with *n *- 1 degrees of freedom.

Further, the standard deviation of the differences of the means is obtained by using one of the two formulas, as appropriate, given below

Applying these formulas to the hospital LOS data, we get, under the *z*-test, that *s_z _*= (30 - 20)/2.326 = 4.299 with 95% CI equal to (1.57, 18.4), and *s_d _*= 33.3. Under the *t*-test, we get that *s_t _*= 4.182 with 95% CI equal to (1.63, 18.4), and *s_d _*= 32.4. These 95% intervals are almost identical. Also both are quite wide, with their lower limits not far from zero days. The possibility of just a minor difference in the hospital LOS for the two groups cannot thereby be excluded.

### Paired binary data

First consider paired binary data under a prospective design. The data format and the cell-wise and marginal proportions are shown in Table 2. The marginal proportions are estimated by

**Table 2:** Paired data and paired proportions

	Alive	Dead	Total	Alive	Dead	Marginal
Alive	*a*	*c*	*a *+ *c*	*π*_11_	*π*_12_	1 - *π *_2_
Dead	*b*	*d*	*b *+ *d*	*π*_21_	*π*_22_	*π*_2_
Total	*a *+ *b*	*c *+ *d*	*n*	1 - *π*_1_	*π*_1_	1.00

#### Risk difference

Researchers usually apply two effect measures for paired prospective designs, risk difference and risk ratio. First consider the former, *δ *= *π*_1 _- *π*_2_. It is estimated by

For this measure, two different variance formulas are usually used, one for null hypothesis testing and the other for confidence interval computation. These formulas are shown in the first two rows of Table 3[11-14].

**Table 3:** Variances for matched pairs comparisons.

Statistic	Variance	Estimate of variance
risk difference (general)*		
risk difference (null)**		
log risk ratio (general) ^†^		
log odds ratio (general) ^‡^		

*Altman et al. [11], p.52.

**Fleiss, Levin and Paik [12], p.375.

^†^Væth and Poulsen [13]

^‡ ^Rothman and Greenland [14], p.286

Suppose the *p*-value for the two-sided *z*-test for risk difference equal to zero is known. This gives the associated standard normal deviate, *z*, from which we determine the null standard error as . With , , and *s*_0 _known, the variance formula from the second row of

Table 3 provides the three equations

Since *a *+ *b *+ *c *+ *d *= *n*, we can solve for *a*, *b*, *c *and *d*. The corresponding solution scheme is in the first row of Table 4. All the numbers are rounded to the nearest integer.

**Table 4:** Reconstructing the 2 × 2 table.

	*b*	*c*
Risk difference		
Risk ratio		
Odds ratio*		
Odds ratio^†^		

Determine the values of *b *and *c *first. Second, for all effect measures,  and *a *= *n *-(*d *+ *b *+ *c*).

*s*_0 _or *s *is estimate of standard error calculated from the *p*-value.

*Estimate  and both proportions known.

^†^Estimate , one proportion , and *p*-value known.

We apply these formulas to the mortality data from Xie et al. Since *p *= 0.023, then *z *= 2.27. From Table 1, we find  = 0.7 - 0.5 = 0.2. Thus, *s*_0 _= 0.2/2.27 = 0.088. Using these with *n *= 60 in the scheme of the first row of Table 4 gives *a *= 10, *b *= 8, *c *= 20, and *d *= 22. Thereby, we learn that the number of pairs in which both subjects died were 2.2 times as many as those in which both subjects remained alive.

After reconstructing the paired 2 × 2 table, we can check whether we are able to reproduce the *p*-value for hospital mortality given in Table 1. Xie et al. used the McNemar test, which has test statistic equal to

to be compared with the standard normal distribution. With *b *= 8 and *c *= 20, we get *T *= 2.268 and *p *= 0.023, which is equal to the *p*-value given by Xie et al.

To get the 95% CI for the risk difference, we first apply the formula in the first row of Table 3 to the reconstructed table to get . Hence the required interval is  which is 0.2 ± 1.96 × 0.084 = (0.035, 0.37).

The number needed to treat (NNT) then is 1/.2 = 5, implying that for every 5 cases with SS & IFI, on average, one additional case will die as compared with those having SS & no IFI. The 95% CI for the NNT, obtained by inverting the CI for the risk difference and reversing the limits, is equal to (3, 29). This interval, like that of the risk difference, is not that precise.

#### Risk ratio

The risk ratio is *θ *= *π*_2 _/*π*_1 _which, for paired prospective data, is estimated by

The variance of the logarithm of  is in the third row of Table 3. Now suppose the *p*-value for the null test of the risk ratio is known. From this, we find the standard normal deviate, *z*.

The associated standard error then is . For this measure, the same variance formula is customarily used for null hypothesis testing and for confidence interval computation. Hence, we directly compute the 95% CI for ln *θ *as , and exponentiate this interval to get the 95% CI for *θ*.

From the variance formula, we construct four simultaneous equations in the same way as for the risk difference. The corresponding solution scheme for *a*, *b*, *c *and *d *in this case is shown in the second row of Table 4. Here also, these numbers are rounded to the nearest integer.

In the above calculations, we assumed that the published *p*-value was calculated using the correct standard error for paired risk ratio. In practice, this may not occur that often, especially when the CI is unreported. If instead the *p*-value was calculated using McNemar's test, we reconstruct the paired 2 × 2 table using the methods for risk difference, and then apply these numbers to get the variance estimate for the log risk ratio in Table 3. We illustrate this approach in a hypothetical situation.

Suppose we need to meta-analyse several studies with the risk ratio as the effect measure. Suppose Xie et al. [10] is one of the selected studies. The above computations then allow us to include it into this meta-analysis even though it does not report the risk ratio, and the risk ratio or its standard error are not directly computable from the paper. Using the paired data table reconstructed above (*a *= 10, *b *= 8, *c *= 20, and *d *= 22), the risk ratio is 30/42 = 0.71, and the standard error of the log-risk ratio is . We exponentiate the logarithmic interval ln (0.71) ± 1.96 × 0.15 to find the 95% CI for the risk ratio as (0.53, 0.95). This CI also indicates a low precision for the study results. The risk ratio and its standard error are now available for the meta-analysis.

#### Odds ratio

Now consider the odds ratio, which is mostly used in case-control studies. The marginal proportions in the paired data table (Table 1) are the probabilities of exposure given the outcome. Such studies usually apply a conditional form of analysis that uses only the discordant pairs whose sum is taken as fixed. The appropriate conditional probabilities for the two types of discordant pairs are *π*_12_/(*π*_12 _+ *π*_21_) and *π*_21_/(*π*_12 _+ *π*_21_). Further, the relevant odds ratio is *ρ *= *π*_21_/*π*_12_, which is estimated by . The (conditional) variance of the logarithm of  is shown in the last row of Table 3.

For the task of reconstructing the paired 2 × 2 table, the odds ratio differs from the other two binary effect measures in an important way. Unlike for the latter, the paired data table can be computed once we know the odds ratio, the marginal proportions and the total number of pairs. This computation scheme appears in the third row of Table 4.

Consider a hypothetical case-control study where the odds ratio is reported , but the CI is not. Also reported are the marginal proportions ( and ) and the total number of pairs (*n *= 80). Using the third row of Table 4, we reconstruct the paired 2 × 2 table:

We then calculate the standard error of the log odds ratio from the last row of Table 3: . The 95% CI for the log odds ratio is ln (2.0) ± 1.96 × 0.32. After exponentiation, we find that the 95% CI for the odds ratio is (1.1, 3.7).

When only one marginal proportion is known, the appropriate *p*-value is also needed. This then allows us to compute the standard error . Like the risk ratio, the same variance formula is customarily used for null hypothesis testing and for confidence interval computation, as given in the last row of Table 3. Using this, we form four simultaneous equations as done earlier. Solving these, we obtain the scheme shown in the last row of Table 4 for reconstructing the needed data table.

Suppose that in the hypothetical example above, only one of the marginal proportions was given  but that *p *= 0.029 was reported. The standard normal deviate here is *z *= 2.18, and the standard error, *s *= 0.318, is obtained in the usual way. The 95% CI is computed as above. To reconstruct the paired 2 × 2 table, we use the last row of Table 4 to get *b *= (1 + 2)/0.318^2 ^≈ 30 and *c *= (1 + 2)/2 · 0.318^2 ^≈ 15. The quantities *d *= 10 and *a *= 25 are obtained as before.

### Impact of *p*-value accuracy

One important consideration is the accuracy of the stated *p*-value. A small absolute change in a small *p *produces a large change in the *z *or *t *deviate. If the *p*-value has not been stated accurately, our computation schemes can yield flawed answers. To get an initial handle on the error involved here, we performed a sensitivity analysis for the data in Table 1. For both comparisons, the *p*-value was changed from 0.015 to 0.024 in increments of 0.001. For hospital LOS, the *z*-test scheme gave 95% CIs ranging from (1.94, 18.06) to (1.32, 18.68), and the *t*-test scheme gave 95% CIs ranging from (2.01, 17.99) to (1.36, 18.64). And for the same range of *p*-values, the computed 95% CI for the risk difference in mortality ranged from (0.048, 0.352) to (0.035, 0.365). These changes are neither dramatic nor practically meaningful. All the intervals are close to the corresponding intervals for the observed *p*-values. Our experience thus far indicates that if the *p*-value is known to two significant digits, the results are sufficiently accurate, and often, one significant digit of accuracy suffices. Detailed simulation studies to resolve the concerns relating to sample size, data structure, and degree of accuracy of the *p*-value are, however, called for.

## Discussion

Our paper gives easy to apply computation schemes to compute confidence intervals and other entities in situations where the needed information is unavailable. For such tasks, our paper is a paired-data counterpart of Pietrantonj [4] that addresses similar problems for unpaired binary data. Note that the computational schemes we give are simpler than their unpaired variants. Further, our paper differs from previous work for paired data cited earlier in that it is not based on the knowledge of a measure of correlation, a rarely reported entity, but requires the *p*-value, a commonly reported item.

The use of our computation schemes is limited by several considerations. First, for continuous data, we need to know if the paired *z *or *t *test was used to obtain the *p*-value. Second, the computation scheme for the risk ratio applies only if the *p*-value was obtained by the use of the risk ratio standard error. This is rarely done. *p*-values for paired binary data are usually computed using a risk difference based (McNemar) test. If we then need the CI for the risk ratio, we use the risk difference scheme to reconstruct the paired data table and obtain the required standard error from this table. Third, as the paired data table can often be reconstituted with the odds ratio, the *p*-value based scheme here will be rarely, if at all, used. We give it for completeness.

Other matters of concern are data structure and sample size. The data are sparse when one discordant pair is much smaller in size than the other, or when both corresponding marginal proportions are near one or zero. Suppose the given *p*-value has been obtained by either the *z*-test or *t*-test for continuous data, or a test based on one of the null standard errors shown in Table 3 for binary data, and the paper notes the actual test used. In each of these cases, the computation scheme we give is valid at all sample sizes and with all data structures, whether sparse, skewed or otherwise. Validity here means that it will give the same confidence intervals as that based on the original data.

Besides these standard tests, a wide variety of other methods for computing *p*-values and CIs for paired discrete and continuous data exist [15,16]. Some of these methods have better statistical properties compared with the others. And some have complex formulas. With sparse data or small number of pairs, different methods may give appreciably different results. For such data, researchers may also use non-parametric or exact methods employing special algorithms that are not amenable to simple formulations [17]. When such methods have been used in a study with small number of pairs or sparse data, and if the CI has not been reported or the data to compute it are not available, developing a procedure to correct the deficiency is not a simple matter. Applying the computation schemes in Table 4 to such data when the *p*-value has been computed using a specialized test is not advisable.

However, simulation studies show that when viewed in terms of their practical impact, the CIs for the paired data risk difference computed by several methods are fairly close to one another when the number of pairs exceeds 50, and provided the data are not too sparse [15,16]. Based on studies of this sort, we recommend our computation schemes even when the *p*-value has been computed using a non-parametric, exact, score, or some other test provided there are more than 50 pairs and the data are not too skewed or sparse. The error involved would be, we suspect, acceptable for the point of view of practical interpretation or incorporation into meta-analysis.

## Conclusion

Confidence intervals allow us to judge the practical implications of a study. For a paired design, the paired data table is more informative than the marginal data summary. The standard error of difference of the means incorporates the correlation between the two paired measures. Without this information, it cannot be computed directly from the standard errors of each measure. In crossover trials, for example, it enables us to examine the presence of a treatment order effect. Reconstructing the paired data table is useful when a study analyzed in terms of the risk difference is not fully reported and incorporating it into a meta-analysis requires the risk ratio or odds ratio. That reconstruction also permits a re-analysis of the data with methods that have better statistical properties, including for performing a meta-analysis.

Our methods are valid for all types of data when the *p*-value has been computed using one of the standard tests we consider, and may have reasonable accuracy even for other tests provided the number of pairs exceeds 50 and the data are not too sparse or skewed. Simulation studies to identify the types of tests, sample sizes, data structures and levels of accuracy of the *p*-value under which they are acceptably accurate are, however, warranted.

Better reporting of paired data is, nonetheless, the optimal solution. When reporting a study, the point estimate, *p*-value, and confidence interval for the main effect measure must be given. For paired binary designs, the full design based 2 × 2 table should be given, and for paired continuous data, the mean and standard deviation for each sample should be augmented by the standard deviation of the differences. Reporting the relevant correlation is an equivalent substitute. Improved reports will make corrective schemes like the ones we give somewhat superfluous. Until the day when such reports are the almost universal norm, however, these schemes will serve a useful purpose.

## Abbreviations

CI: confidence interval; IFI: invasive fungal infection; LOS: length of stay; NNT: number needed to treat; SS: severe sepsis.

## Competing interests

The authors declare that they have no competing interests.

## Authors' contributions

KFH conceived the study and wrote the initial and final drafts. MWF performed the literature survey, did additional computations, gave comments and made additions to the final draft. Both authors read and approved the final manuscript.

## Pre-publication history

The pre-publication history for this paper can be accessed here:

http://www.biomedcentral.com/1471-2288/11/66/prepub
